# Supplementary material for: SMART (artificial intelligence enabled) DROP (diabetic retinopathy outcomes and pathways): Study protocol for diabetic retinopathy management
Source: PLoS One. 2025 May 19;20(5):e0324382. doi: 10.1371/journal.pone.0324382 (PMC12088010; doi:10.1371/journal.pone.0324382)
Supplement: Attachment — Submitted filename: Response to reviewers.docx [file pone.0324382.s002.docx]

1. Please ensure that your manuscript meets PLOS ONE's style requirements, including those for file naming. The PLOS ONE style templates can be found at

<https://journals.plos.org/plosone/s/file?id=wjVg/PLOSOne_formatting_sample_main_body.pdf> and

[https://journals.plos.org/plosone/s/file?id=ba62/PLOSOne_formatting_sample_title_authors_affiliations.pdf](https://track.editorialmanager.com/CL0/https:%2F%2Fjournals.plos.org%2Fplosone%2Fs%2Ffile%3Fid=ba62%2FPLOSOne_formatting_sample_title_authors_affiliations.pdf/1/010f0194820e5569-f0e24ab8-1b48-4103-9a3e-623304d24abc-000000/reU8pHfMZGnERzESh7eDoUWL9xm1BV3sKEIhqzQHFeM=194)

**Response: Suggested changes have been made in the revised manuscript as per the guidelines.**

2. Please note that funding information should not appear in any section or other areas of your manuscript. We will only publish funding information present in the Funding Statement section of the online submission form. Please remove any funding-related text from the manuscript.

**Response: Suggested changes have been made in the revised manuscript.**

3. Your ethics statement should only appear in the Methods section of your manuscript. If your ethics statement is written in any section besides the Methods, please move it to the Methods section and delete it from any other section. Please ensure that your ethics statement is included in your manuscript, as the ethics statement entered into the online submission form will not be published alongside your manuscript.

**Response: Suggested changes have been made in the revised manuscript.**

4. We note that Figure 2 in your submission contain map/satellite images which may be copyrighted. All PLOS content is published under the Creative Commons Attribution License (CC BY 4.0), which means that the manuscript, images, and Supporting Information files will be freely available online, and any third party is permitted to access, download, copy, distribute, and use these materials in any way, even commercially, with proper attribution. For these reasons, we cannot publish previously copyrighted maps or satellite images created using proprietary data, such as Google software (Google Maps, Street View, and Earth). For more information, see our copyright guidelines: <http://journals.plos.org/plosone/s/licenses-and-copyright>.

**Response: We have updated Figure 2 to use OpenStreetMap, which is ODbL-licensed and meets PLOS requirements. The figure now includes proper attribution: "Map data © OpenStreetMap contributors, licensed under ODbL."**

5. Please ensure that you refer to Figure 1 in your text as, if accepted, production will need this reference to link the reader to the figure.

**Response: Suggested changes have been made in the revised manuscript.**

**Figure 1 is supporting information file (SPIRIT Template for Clinical Trial) for the study. This has been cited in the revised manuscript under supporting files list.**

6. Please include a separate caption for each figure in your manuscript.

**Response: Suggested changes have been made in the revised manuscript.**

7. Please include captions for your Supporting Information files at the end of your manuscript, and update any in-text citations to match accordingly. Please see our Supporting Information guidelines for more information: <http://journals.plos.org/plosone/s/supporting-information>.

**Response: Suggested changes have been made in the revised manuscript.**

Additional Editor Comments:

Dear Authors
The study protocol described in the manuscript requires further specifications to make it more objective. Please go through the reviewers' comments and modify the manuscript accordingly.

**Response: We thank the reviewers for their valuable comments, all of which have been addressed in the revised manuscript.**

PONE-D-24-54914

SMART (artificial intelligence enabled) DROP (Diabetic Retinopathy Outcomes and Pathways): Study Protocol for Diabetic Retinopathy Management

PLOS ONE

Reviewer #1: The study is relevant and addresses a critical public health issue by focusing on early detection of diabetic retinopathy (DR) and the potential impact of systematic screening.
Objectives are clearly stated and include outcomes such as earlier detection, reduced visual impairment, and a comparison of different screening settings.
The inclusion of AI accuracy as a secondary outcome is novel and could inform future AI implementations in healthcare.

Sample Size and Sampling
• The sample size appears to be based on prevalence but may not account for the need to evaluate AI diagnostic performance (sensitivity/specificity). Please include a separate calculation for diagnostic evaluation.

**Response: We have included separate sample size calculation for diagnostic performance of AI in the revised manuscript. (Page no:15 Line no:337-342 )**

• Clarify the sampling method (e.g., random or convenience sampling) and ensure it minimizes bias. For example, confirm whether recruitment will be balanced across physician clinics and eye care settings.

**Response: The study will employ a convenience sampling method, recruiting all consecutive eligible individuals with diabetes mellitus (DM) visiting physician clinics and eye care settings who meet the standard inclusion and exclusion criteria and provide informed consent. While convenience sampling is utilized, potential sources of bias include selection bias, site-specific variation, and differential access to care. To mitigate these, systematic recruitment will be ensured by enrolling all eligible patients consecutively at each site, reducing the likelihood of preferential selection. Recruitment will take place across multiple physician clinics and eye care settings to improve representativeness and minimize site-specific variation. Differential access to care will be monitored by assessing demographic and clinical characteristics of enrolled participants, allowing adjustments to recruitment strategies if needed. To prevent observer bias, standardized screening protocols and training for healthcare personnel will be implemented, ensuring consistency in recruitment and data collection. These measures aim to enhance the validity and generalizability of the findings while acknowledging the inherent limitations of convenience sampling.**

**Above clarification has been included in the revised text.(Page no:13-14, Line no: 307-321)**

Statistical Analysis:

Provide more details on the statistical methods to evaluate key outcomes.
• Use sensitivity, specificity, and receiver operating characteristic (ROC) curve analysis to validate the AI tool.

**Response: We have included above parameters for estimating diagnostic accuracy of AI. The table has been updated to include ROC outcome measure for diagnostic performance in the revised manuscript. Accuracy of AI-enabled cameras: Taking the grading of images done by the experts as the ground truth, we will estimate the accuracy of the AI- enabled cameras (operated by a non- ophthalmic technician) using standard measures such as sensitivity, specificity, and area under the ROC (Receiver Operating Characteristic). (Page no:17 Line no:395 and Page 18,Table )**

• Include multivariable models to adjust for confounders like age, diabetes duration, and baseline visual acuity.

**Reply: Suggested changes have been incorporated in the revised manuscript. (Page no: 13,Line no: 298-306)Multivariable regression models will be employed to adjust for potential confounders, including age, diabetes duration, baseline visual acuity, and other relevant factors. These models will allow for a more accurate assessment of the relationships between the intervention and key outcomes, such as disease staging at diagnosis, referral rates, and long-term visual outcomes, by accounting for the influence of these confounders. Both linear and logistic regression models will be used, depending on the nature of the outcome variable, to ensure robust and reliable results.**

Ethical Considerations:
• Describe how participant data will be anonymized and securely managed.
• Detail how patients who withdraw or are lost to follow-up will be handled in the analysis.

**Reply: Suggested changes have been incorporated in the revised text. (Page no:19-20, Line no: 412-433).All participant data will be anonymized to ensure confidentiality. Unique identification codes will be assigned to participants, and no personally identifiable information (PII) will be linked directly to the dataset. Data will be securely stored in encrypted digital formats on password-protected servers with access restricted to authorized personnel. Hard copies, if any, will be stored in locked cabinets within secure facilities. Any data transfer between study sites will be conducted using secure, encrypted channels to maintain data security.**

**Participants who withdraw from the study will have their data excluded from subsequent analyses unless explicit consent is provided for its continued use. Patients lost to follow-up will be included in an intention-to-treat analysis to minimize bias and preserve the benefits of systematic recruitment. Sensitivity analyses will be performed to evaluate the potential impact of missing data. Where applicable, missing data will be addressed using appropriate imputation methods, such as multiple imputation or the last observation carried forward, to ensure the robustness and validity of the study findings.**

Reviewer #2: I have several concerns regarding the study's methodology and statistical approach:

I am not clear about rationale for the association between the intervention and improved AI prediction accuracy. Which AI model will be employed, and how will the authors test and validate its performance?

**Response: The project employs two fundus camera models with inbuilt AI algorithms. Non-mydriatic fundus photography of both eyes will be performed using AI-integrated retinal cameras (Forus, 3 Nethra Classic HD with AI Integration in two facilities, and Remidio, Fundus on Phone with AI integration, in two facilities). All images are sent by cloud transfer to a central image reading centre, where in the images are manually graded. Taking the grading of images done by the experts at the image reading centre as the ground truth, we will estimate the accuracy of the AI- enabled cameras (operated by a non- ophthalmic technician) using standard measures such as sensitivity, specificity, and area under the ROC (Receiver Operating Characteristic). (Page no:9 Line no:210-215, Page no:17 Line no:395 and Page 18,Table )**

In line 213, it seems that no technician will confirm or rule out an AI-determined ‘no DR diagnosis.’ If this is the case, how will the specificity of the model be evaluated?

**Response: The proposed methodology ensures timely referral of patients and aims to minimize false positive referrals by the AI system. To evaluate the specificity of the model, all fundus images will be graded at a central image reading center by expert graders. The manual grading results from the central reading center will serve as the ground truth to accurately determine the sensitivity and specificity of the AI system for detecting diabetic retinopathy. This approach ensures that the performance metrics of the AI model are validated against a reliable and expert-verified standard.**

Line 242, is N=25 sufficient? How was this sample size determined?

**Response: The visual acuity tool used in the study (Peek Vision) has been globally validated in multiple studies and effectively utilized by technicians and field workers in diverse settings.[33, 34]The sample size of N=25 patients (50 eyes) is intended for pilot testing following standardized training. This pilot will serve as an initial assessment of the reliability and accuracy of visual acuity estimation using the mobile VA application by trained technicians. The pilot testing results will be used to validate the training protocol and identify any gaps requiring refinement.**

**To ensure ongoing reliability, periodic audits will be conducted during training refresher programs, where technicians' performance will be re-evaluated. Additionally, comparisons of visual acuity estimates from referred patients will be performed to assess consistency over time. This iterative approach ensures that the quality of visual acuity measurements is maintained throughout the study. (Page no:11,Line no: 253-264)**

The statistical analysis appears to address only one outcome. How will other outcomes be accounted for, and which statistical tests will be used for group comparisons?

**Response: The suggested changes have been included in the revised manuscript.**

**Sample size for other outcomes such as AI diagnostic accuracy and patient satisfaction has been included in the revised text. Page no:15, Line no:337-358 )**

**Multivariable regression models will be employed to adjust for potential confounders, including age, diabetes duration, baseline visual acuity, and other relevant factors. These models will allow for a more accurate assessment of the relationships between the intervention and key outcomes, such as disease staging at diagnosis, referral rates, and long-term visual outcomes, by accounting for the influence of these confounders. Both linear and logistic regression models will be used, depending on the nature of the outcome variable, to ensure robust and reliable results. Additional statistical tests, such as chi-square tests for categorical outcomes and t-tests or ANOVA for continuous outcomes, will be employed as appropriate. (Page no:13, Line no:298-306 )**

What method was used to calculate the sample size, and what effect size was assumed in this calculation?

**Response: The sample size was calculated using a difference in proportions method to detect meaningful differences in the staging of sight-threatening diabetic retinopathy (STDR) between the intervention and standard-of-care (SOC) arms at their first ophthalmology visit. An effect size was assumed based on historical data, with an estimated 50% prevalence of STDR in the SOC arm and a lower prevalence of approximately 10% in the intervention arm at the time of initial screening. However, to account for non-compliance, the prevalence of STDR among patients completing their first ophthalmology visit in the intervention arm was adjusted to 25%. The calculat362-367ion assumed a power of 80% and a Type-I error rate of 5%.**

Reviewer #3: The study addresses a critical public health challenge, particularly in low-resource settings like India, where the burden of diabetes and DR continues to rise. By leveraging innovative technologies and multidisciplinary collaboration, the proposed study has potential to significantly improve access to timely DR diagnosis, reduce visual impairment and inform cost effective health care strategies. Overall, the manuscript is well structured and demonstrates a strong commitment to research rigor and public health relevance. Following are areas that could benefit from further elaboration:

Introduction:
1. Consider emphasizing the significance of cost effectiveness more explicitly as that is one of the study objectives.

**Response: The suggested changes have been included in the revised manuscript.(Page no: 16,Line no: 362-367 )The evidence on cost-effectiveness of screening for DR using digital technologies is mixed. For instance** [**Avidor et al. (2020)**](https://link.springer.com/content/pdf/10.1186/s12962-020-00211-1.pdf) **found that the results varied from not being cost-effective to being cost-effective to being cost-saving based on the context and the frequency and modality of the screening and the use of telemedicine. Given the resource constraints in an LMIC like India, it is imperative that the AI solutions proposed are cost-effective (preferably cost-saving) if they are to be adopted at scale.**

Methods:
2. Include a brief justification for the choice of study sites, addressing their relevance and representativeness for scaling the findings.

**Response: The suggested changes have been included in the revised manuscript.(Page no:8,Line no: 177-185 ).**

**The LV Prasad Eye Institute is a leading tertiary eye care center with advanced infrastructure for diabetic retinopathy diagnosis and treatment, making it an ideal reference for validating the intervention. The IDEA Clinics are strategically located in urban areas with a high burden of diabetes and diabetic retinopathy, ensuring the recruitment of a relevant and representative population. These clinics provide comprehensive diabetes care and offer an optimal setting to test the integration of AI-enabled diabetic retinopathy screening within existing care pathways. The combination of these sites allows for the evaluation of the intervention's feasibility and scalability in diverse healthcare settings, supporting its potential for broader implementation.**

3. Provide information on the AI system, such as its development, training dataset characteristics and prior accuracy metrics, if available.

**Response: Suggested changes with supporting published literature has been included in the revised manuscript.(Page no:9 Line no: 212-215 ).**

4. Expand on how patient satisfaction will be assessed including details on survey tools or scales to be used.

**Response: The suggested changes have been included in the revised manuscript.(Page no:15, Line no:351-358).**

**To assess patient satisfaction, a sample size of 316 patients will be selected from both the AI-enabled and routine eye care pathways, calculated to detect a difference in satisfaction rates (80% vs. 65%) with a 95% confidence level and a margin of error of ±5%. Random sampling will be employed to ensure unbiased selection of participants from each pathway. This method will involve assigning unique identifiers to eligible patients and using a randomization tool to select participants, ensuring the sample is representative of the study population.**

**Patient satisfaction will be assessed using a structured survey that includes validated tools like the Patient Satisfaction Questionnaire (PSQ-18) and custom questions specific to the AI-enabled screening program. The survey will use a Likert scale (e.g., 1 = very dissatisfied to 5 = very satisfied) to evaluate domains such as accessibility, process experience, and outcome satisfaction. Surveys will be administered after screening and follow-up visits, either digitally or on paper, to capture immediate and longitudinal feedback. Responses will be anonymized and analyzed to assess overall satisfaction and identify areas for improvement.**

5. Provide a brief overview of data protection measures, including encryption methods, user access protocols and compliance with relevant data protection regulations.

**Response: The suggested changes have been included in the revised manuscript.(Page no:19, Line no: 421-426 ). All participant data will be anonymized to ensure confidentiality. Unique identification codes will be assigned to participants, and no personally identifiable information (PII) will be linked directly to the dataset. Data will be securely stored in encrypted digital formats on password-protected servers with access restricted to authorized personnel. Hard copies, if any, will be stored in locked cabinets within secure facilities. Any data transfer between study sites will be conducted using secure, encrypted channels to maintain data security.**

Discussion:
6. Critically analyse potential challenges in implementation, such as variability in AI performance or patient adherence, and discuss possible mitigation strategies.

**Response: Suggested changes have been incorporated in the revised manuscript. (Page no: 21-22,Line no:468-491 )**

**The implementation of the study may face challenges such as variability in AI performance, patient adherence to referrals and follow-up, operational issues across multiple sites, patient acceptance of AI tools, and resource constraints for scale-up. To address these potential challenges, we plan periodic recalibration of the AI system, standardized technician training, and regular quality audits to ensure consistent performance. Strategies to improve patient adherence will include employing navigators, simplifying referral pathways, and providing education on the importance of timely care. Operational issues will be managed through centralized coordination, cloud-based data management, and regular equipment maintenance. Patient trust in AI tools will be fostered through education, validation of AI outputs by experts, and sharing of success stories. Resource constraints will be mitigated by phased implementation in high-burden areas and leveraging public-private partnerships. Additionally, ensuring adherence to the Standard Operating Procedures (SOPs) in a private clinic chain—where business performance may take precedence—can be challenging, as operational priorities may not always align with clinical protocols. Furthermore, monitoring compliance across multiple locations is complex due to variations in workflow, staff engagement, and resource availability. Adherence to SOPs in a private clinic chain with business priorities will be ensured by aligning protocols with clinic goals, incentivizing compliance, and integrating them into existing workflows. Stakeholder engagement and automated compliance tracking will further support adherence. Monitoring across multiple locations will be managed through real-time dashboards, automated alerts, remote audits, and periodic site visits. Local champions will be designated in each clinic to enhance accountability and streamline implementation. These measures are expected to support effective and scalable implementation of the study.**

7. Highlight the policy-level implications of the study findings, particularly how they could influence national programs such as Ayushman Bharat.

**Response: Suggested changes have been incorporated in the revised manuscript.(Page no:23, Line no: 500-506).The study findings could significantly influence national programs like Ayushman Bharat by supporting the integration of AI-enabled DR screening into routine care. Evidence on the accuracy, cost-effectiveness, and streamlined referral mechanisms could optimize resource use and expand access. Additionally, the results may inform policies on reimbursing AI-assisted screening by trained non-medical technicians, enhancing coverage and early detection of DR nationwide.**

8. The authors have just listed the limitations of the study, which is important. Consider also discussing how these limitations might affect the generalizability of the findings. Additionally, suggest ways to minimize the potential selection biases introduced by including only private clinics and address image quality issues due to reliance on non-mydriatic cameras.

**Response: Suggested changes have been incorporated in the revised manuscript.(Page no:22 -23, Line no: 492-499 )**

**Given that private providers manage 70-80% of all patients, with an even higher proportion in advanced ophthalmologic conditions like DR, the findings are highly relevant to real-world care delivery. However, the study’s reliance on private healthcare clinics may limit generalizability by excluding patients unable to afford screening. To reduce selection bias, future studies should include public healthcare settings. Non-mydriatic fundus photography may impact image quality and DR detection accuracy; this can be mitigated through enhanced technician training, image quality audits, and advanced AI algorithms. These steps can improve the applicability of findings across diverse settings.**
