# Supplementary material for: SMART (artificial intelligence enabled) DROP (diabetic retinopathy outcomes and pathways): Study protocol for diabetic retinopathy management
Source: PLoS One. 2025 May 19;20(5):e0324382. doi: 10.1371/journal.pone.0324382 (PMC12088010; doi:10.1371/journal.pone.0324382)
Supplement: Attachment — Submitted filename: Reply to reviewers comments.docx [file pone.0324382.s003.docx]

Response to Reviewers

Reviewer #1: The revisions have significantly improved the clarity and methodological rigor of your manuscript. The inclusion of a separate sample size calculation for evaluating AI diagnostic performance strengthens the study’s statistical foundation.
The addition of sensitivity, specificity, and ROC curve analysis for AI validation ensures a more robust assessment of its effectiveness.

**Response : Thank you for your valuable comments.**

The revised statistical analysis section now includes appropriate multivariable regression models to adjust for confounders, improving the validity of findings.
While cost-effectiveness has been emphasized, consider including a clearer breakdown of expected cost components (e.g., personnel training, AI maintenance) to strengthen this section.

**Response : Thank you. Suggested changes have been included in the revised manuscript. Page 16, Line 372-375.**

Image Quality in Non-Mydriatic Photography: While the manuscript acknowledges limitations related to image quality, additional discussion on how AI adjustments or technician training will mitigate this issue would be beneficial.

**Response : Thank you. Suggested changes have been included in the revised manuscript. Page 21,Line 471-477**

Reviewer #2: All my comments are addressed.

**Response : Thank you for your valuable comments.**
