# Supplementary material for: SMART (artificial intelligence enabled) DROP (diabetic retinopathy outcomes and pathways): Study protocol for diabetic retinopathy management
Source: PLoS One. 2025 May 19;20(5):e0324382. doi: 10.1371/journal.pone.0324382 (PMC12088010; doi:10.1371/journal.pone.0324382)
Supplement: Attachment — Submitted filename: Response to reviewer comments.docx [file pone.0324382.s001.docx]

PONE-D-24-52472 dated 22^nd^ November 2024
SMART (artificial intelligence enabled) DROP (Diabetic Retinopathy Outcomes and Pathways): Study Protocol for Diabetic Retinopathy Management
PLOS ONE

Dear Dr. Rani,

Thank you very much for submitting your manuscript to PLOS ONE, and for responding to our recent requests regarding your submission. After careful evaluation, we have decided that your submission does not meet our publication criteria and must be rejected.

Based on our evaluation of your manuscript and the information you provided, we do not feel that your submission meets our ethical requirements for human subjects research submissions. PLOS ONE requires that research meets all applicable standards for the ethics of experimentation and research integrity (<http://journals.plos.org/plosone/s/human-subjects-research>). We reserve the right to reject any submission that does not meet our internal ethical standards, which in some cases are more stringent than local ethical standards.

We recognize that you obtained ethical approval from your local ethics committee after you began your study: your ethics approval document from the Ethics Committee of  L V Prasad Eye Institute is dated 6 October 2023, while participants were recruited beginning on 29 March 2023. You also state that informed consent was obtained from all participants, which indicates that the samples were prospectively collected for research purposes. For human subjects research, PLOS ONE requires that authors obtain ethics committee approval before beginning the study, and we do not accept retrospective ethical approval.

As a result of these concerns we cannot consider the manuscript for publication at PLOS ONE. I am sorry that we cannot be more positive on this occasion, but hope that you understand the reasons for this decision.

Yours sincerely,

Yuvaranjini Mallikeswaran

Editorial Office

PLOS ONE

**Response to Editor’s Comments**

Dear Editor,

Thank you for your review and feedback on our manuscript, **"SMART (Artificial Intelligence Enabled) DROP (Diabetic Retinopathy Outcomes and Pathways): Study Protocol for Diabetic Retinopathy Management."**

We sincerely regret the typographical error in our initial submission that led to confusion regarding the study timeline. We would like to clarify that participant recruitment for this study began on **March 29, 2024**, not March 29, 2023, as was mistakenly stated. This correction has been updated in the manuscript (Page **6, Line 229-231).**

As noted in our revised submission support files, ethical approval for this study was obtained on **October 6, 2023**, from the Ethics Committee of L V Prasad Eye Institute, which was well in advance of the actual commencement of patient enrolment. All participants provided informed consent after the ethical approval was granted.

We appreciate your careful review process and sincerely apologize for any confusion the date discrepancy may have caused. We hope that this clarification allows for reconsideration of our submission.

Thank you for your time and understanding.

Sincerely,
Padmaja Kumari Rani, MS, FRCS, FICO, FNB (Retina)
Network Associate Director, Department of Teleophthalmology
L V Prasad Eye Institute, Hyderabad, India
Phone: +91 040-68102230 | Email: rpk@lvpei.org
